# Supplementary material for: Are carbon emissions trading and green financial instruments synergistic? -Comprehensive quantitative research based on content analysis
Source: PLoS One. 2024 Mar 7;19(3):e0298601. doi: 10.1371/journal.pone.0298601 (PMC10919601; doi:10.1371/journal.pone.0298601)
Supplement: S1 Data — (ZIP) [file pone.0298601.s001.zip › Data/地级市Z_F工作报告.zip!/╣π╬≈/╙±┴╓╩╨/└┤╫╘╣1⁄2╓┌║┼í╛┬φ┐╦╩2╛▌═°í┐_2009.docx]

各位代表：

现在，我代表市人民政府向大会报告工作，请予审议，并请市政协委员和其他列席会议的同志提出意见。

一、2008年工作回顾

2008年是极不平凡的一年，大事多、喜事多、难事多。面对机遇和挑战，我们坚持以科学发展观为统领，认真学习贯彻胡锦涛总书记等中央领导到广西考察指导工作时的重要讲话精神，在自治区党委、政府的正确领导和市委的坚强领导下，牢牢把握多区域合作机遇，积极应对各种挑战，着力转变发展方式，推动经济持续较快增长，注重改善民生，促进社会和谐，取得了建设富裕文明和谐新玉林的新成就。

经济平稳较快发展，综合实力稳步提升。初步统计，2008年全市生产总值达605.92亿元，增长12.9%，连续5年实现两位数增长。规模以上工业实现增加值114.51亿元，增长17.4%。社会消费品零售总额219.51亿元，增长23.5%。外贸进出口总额4.42亿美元，增长19.5%。固定资产投资完成290.69亿元，增长26%。财政收入47.75亿元，增长17.4%。发展方式不断转变。万元GDP能耗约1.37吨标准煤，同比下降5%；规模以上工业万元增加值能耗1.94吨标准煤，同比下降7.14%；化学需氧量削减2.26万吨；二氧化硫削减0.86万吨。城镇生活垃圾处理设施建设进展顺利，市污水处理厂一期工程建成并试水运行；各县（市、区）污水处理项目全面提速。深入实施第四轮科技创新计划，科技对经济增长的贡献率持续提高。

结构调整更加合理，产业结构日趋优化。投资、消费、出口均保持较快增长，三次产业全面发展，比重调整为24.8:38.2:37。制定实施《关于建设广西现代农业和农业产业化示范市的决定》，农业发展水平继续保持全区领先地位。全年实现农林牧渔业总产值256.66亿元，同比增长5.9%。特色农业加快发展，被自治区政府授予广西农业优势特色产业（优质粮食）一等奖；农业产业化水平持续提高，全市有农业产业化经营组织2110个，其中经工商登记的专业合作社777家，数量居全区首位，带动农户54.36万户，占全市农户总数的46%。“强柴兴玉”战略取得新成效，玉柴集团全年营业收入208.4亿元，增长13.5%，产业发展、市场布局、研发水平取得新突破，实现“三年再造一个玉柴”目标，玉柴品牌获评为中国十大自主创新品牌之一。工业发展再上新台阶，全年完成工业增加值202.68亿元，同比增长15.2%；坚持品牌引领、集聚发展，产业集群不断发展壮大，机械、水泥陶瓷、健康、服装皮革和电子信息五大产业集群规模以上工业企业共实现增加值75.4亿元，增长17.1%，对规模以上工业增长的贡献率达64.3%；园区经济迅速发展，成为工业经济的强劲增长点。服务业保持较快发展势头，全年第三产业增加值224.1亿元，增长15.2%。

城市空间不断拓展，集聚功能日益完善。玉-北-福一体化和县城基础设施建设大会战取得新成效，新区开发全面提速，旧城改造和谐推进，公共设施不断完善，产业布局同步铺开，实现了城市发展量的扩张与质的同步提升。玉林市污水处理厂一期工程等一批项目竣工投入使用；商务大厦、迎宾大道一期工程、为民路景观大道、南流江改造二期工程、玉林城区垃圾收运系统等一批公共设施建设加快推进，新建、改造、完善了一批城市道路和小街小巷。大力创建广西园林城市，深入实施“城乡清洁工程”，城市品位进一步提高，市本级荣获第六届广西市容“南珠杯”竞赛B类城市特等奖，参赛的北流、容县、陆川、博白、兴业全部获奖。全年市政公用设施建设投入达12亿元，增长1.03%。房地产开发投资36亿元，增长26%。玉林城区建成区面积扩大到52平方公里，全市城镇化率提高到35.4%。

项目投资提速增效，发展后劲显著增强。重大项目建设取得新突破。去年，全市统筹推进的新开工和续建重大项目433个，完成投资178亿元。北流海螺水泥余热发电项目、三环集团特种陶瓷项目、玉柴柴油机及挖掘机零配件项目、龙潭至山口一级公路、陆川华润水泥有限公司日产4500吨水泥熟料新型干法水泥生产线、玉林至铁山港高速公路等一批项目相继开工；洛湛铁路玉林段等一批工程建成在即；岑兴高速公路、燕京啤酒玉林分公司三期工程、玉柴华原空气滤清器生产项目等一批重大项目竣工投入使用。

改革开放加快推进，区域合作水平拉升。重点领域改革取得新进展，国有集体企业改革稳步推进，财税、投资、价格改革和医疗卫生体制改革取得阶段性成效。制定实施《关于建设广西非公经济示范市的决定》，进一步激发了非公经济发展活力。开放合作不断深化，第五届玉博会圆满成功，签订106个合同项目，合同投资额190.2亿元，比上届增长5.32%；与珠三角、长三角等区域合作进一步拓展，与梧州、贵港、贺州共同打造广西东部产业转移示范区。融入北部湾经济区步伐加快，成立了龙潭产业园工作机构，进一步完善了建设规划；与北海市签订了铁山港东岸开发协议和铁山港（龙潭）组团合作规划建设框架协议，加快交通、物流、产业等方面与北部湾地区全面对接；龙潭产业园入园企业已达12家，广西银亿科技矿冶有限公司建成试产，总投资达200多亿元的再生资源进口加工利用项目已通过环保部审批。积极开展招商引资，推进招商引资项目大兑现工作，招商引资和承接产业转移工作取得新成效。去年，外商直接投资按商务部口径统计2264万美元，增长63.5%；招商引资新签合同项目425个，合同金额257.19亿元，引进区外境内实际到位资金超过180亿元。

民生保障持续改善，发展成果惠及群众。加大对社会事业的投入，年初承诺的为民办实事10项工程提前1个月完成。继续巩固提高“两基”工作，全市农村义务教育阶段学生和城市低保家庭学生享受免费教科书；对98万名义务教育阶段学生全部免除学杂费，20.5万名家庭经济困难寄宿生获得补助；资助贫困大学新生5098名，发放助学金457.71万元。职业教育攻坚取得新进展，民办职业教育继续保持全区领先地位。城乡公共文化服务体系建设进一步加强，竞技体育和全民健身活动取得新成效。城乡医疗惠民工程成效显著，新型农村合作医疗覆盖全市所有县(市、区)，469.5万农民参合，参合率85.6%。农村人口饮水安全工程全面完成，解决10.99万人饮水安全问题。人口计生事业健康发展，人口自增率控制在10‰以内。农村最低生活保障水平稳步提高，全市享受农村低保人数达22.25万人，占农村人口的4.07%；发放低保金9213万元，实现应保尽保。全市廉租房建设工程开工建筑面积1.48万平方米，占任务的147.7%。农村贫困残疾人危房改造工程、农村公路建设工程建设任务全面完成。广播电视村村通工程进展顺利，全市338个行政村1731个自然村实现光纤联网。城乡电网建设进一步完善。全市新建沼气池12543座，占全年任务的102.3%。完成22座病险水库除险加固。玉林城区道路硬化绿化亮化工程全面完成，完成7条道路和86条小街小巷硬化改造。实施“万村千乡市场建设工程”，新建的300家农家店全部通过验收。市本级财政安排专项资金210万元，各级各部门积极筹资，大幅度增加对老区建设的投入，老区群众生产生活条件不断改善。城乡居民生活水平持续提高。全年城镇居民人均可支配收入14156元，增长16%；农民人均纯收入4123元，增长16.6%。社会保险覆盖面不断扩大，五项社会保险参保人数96.05万人，发放保险金6.1亿元。扶贫开发工作和第二批贫困村“整村推进”工作扎实展开。水库移民工作取得新成效。安全生产、产品质量和食品药品安全工作得到有效加强。“两会一部”股金风险得到有效化解。社会矛盾预防和化解机制逐步完善，严厉打击各种刑事犯罪活动，维护了社会和谐稳定。

政府建设明显加强，服务效能不断提高。去年以来，我们以开展继续解放思想大讨论活动为契机，不断加强政府自身建设。认真执行市人大及其常委会的各项决议决定，自觉接受市人大及其常委会的法律监督和工作监督。加强同人民政协的联系，支持政协委员发挥参政议政作用，顺利完成人大代表建议和政协委员提案的办理工作。广泛听取各民主党派、工商联、无党派人士的意见，支持工会、共青团、妇联等人民团体更好发挥作用。依法治市扎实推进，普法、法律监督和法律服务迈出新步伐。进一步转变政府职能，精简和规范行政审批，加快推进决策科学化、民主化。村民（居民）自治和政务（村务、厂务）公开取得新进展。政务中心建设深入推进，七个县（市、区）全部建立政务服务中心。电子政务建设取得新成效，中国玉林政府网站荣获“中国地方政府网站创新品牌奖”和“全国地级市政府网站群管理进步奖”。政府廉政建设持续加强，政府执行力、公信力进一步提高。

此外，经全市上下团结协作，我市顺利完成自治区成立50周年各项庆典活动，得到了自治区的肯定。人事人才、机构编制、机关事务、应急管理、新闻出版、发展研究、外事侨务、对台事务、民族宗教、旅游、监察、金融、审计、物价、统计、工商、质监、税务、通讯、无线电管理、海关、检验检疫、粮食、农机、供销、二轻、气象、水文、防震、地矿、人防、房改、住房公积金管理、档案、修志等各项工作取得新成效，关心下一代、老龄、残疾人事业健康发展。

各位代表！以上成绩，来之不易。去年，我市先后遭受了历史罕见的低温雨雪冰冻特大灾害和洪涝灾害，受灾人口达225万人次，直接经济损失17.6亿元。全市各级各部门和广大干部群众在市委领导下，全面动员，积极应对，奋起抗灾，共投入灾民生活安排资金3465万元，防洪抗灾资金5200万元；同时，积极支持四川地震灾区，全市共捐款（物）折合人民币4850万元。去年，我们还积极应对世界金融危机对经济发展的冲击，认真落实中央、自治区扩大内需的政策措施，出台了《关于应对国际金融危机确保全市工业平稳发展的若干措施》，加大了对中小企业的扶持；抓住中央扩大内需重要机遇，全力争取中央资金支持，共获得2008年中央新增1000亿元投资的2.27亿元。

各位代表！以上成绩，是自治区党委、政府和市委正确领导、市人大及其常委会监督支持、人民政协民主监督和各民主党派、工商联积极参政议政的结果，是全市人民同心同德、顽强拼搏、克难攻坚的结果。在此，我代表市人民政府，向全市人民，向驻玉部队、武警官兵、公安干警和离退休老同志，向各民主党派、工商联、人民团体和社会各界，向所有关心、支持玉林建设发展的港澳台同胞及海内外朋友们表示衷心的感谢，并致以崇高的敬意！

在看到成绩的同时，我们也清醒地认识到，今年我们面临的经济形势异常复杂严峻，可能是新世纪以来我市经济最为困难的一年。受国际金融危机影响，我市主要经济指标增幅回落，保增长压力加大；节能减排形势严峻，发展方式粗放，促转型任务繁重；中小企业抗风险能力差，部分行业和企业由于需求缩减、资金短缺、成本上升等原因，生产经营困难加剧；外出务工返乡农民工增加，就业形势更趋严峻，城乡居民增收难度加大。此外，干旱等不可预知的自然因素还有可能影响粮食生产稳定和农民持续增收。政府工作中还存在一些问题和不足，历史遗留问题比较突出，严峻经济形势有可能诱发潜在社会矛盾，和谐社会建设任重道远。我们一定高度重视这些困难和问题，切实增强忧患意识和责任意识，把困难估计得更充分一些，把措施考虑得更周密一些，把工作做得更扎实一些。

二、2009年发展形势和目标任务

2009年是新中国成立60周年，是我市实现“十一五”规划目标和实施科学发展三年计划的攻坚年，也是全面开展深入学习实践科学发展观活动、促进科学发展上水平、抢抓机遇加快发展的重要一年。做好今年的政府工作，具有十分重要的意义。

辩证地看，今年，我们面对的挑战前所未有，面临的机遇也前所未有，有利条件和积极因素更是前所未有：一是中央应对国际金融危机一系列得力有效的决策部署，为全国实现平稳较快发展提供了坚强支撑。国际金融危机虽然使我国经济受到一定影响，但我国经济发展的基本面和长期趋势没有改变，我区保持良好发展态势没有改变，玉林加快发展的总体趋势也不会改变。二是改革开放30年的发展建设，我市综合经济实力不断增强，打下了良好的体制、物质和技术基础，具备了较强的风险抗御能力。三是国家深入实施西部大开发战略，中央将专门出台加快广西发展的政策措施，这将为我市加快发展提供强有力的政策支持。四是广西北部湾经济区正迎来加快开放开发的新高潮，我市作为北部湾经济区“4+2”城市，将借助这一重要平台推进新一轮的大发展。五是我市正处在工业化、城镇化快速发展阶段，拉动经济发展的投资和消费潜力很大，加快发展的动力较强。

正确分析形势，充分估计困难，目的在于统一认识，坚定信心。黄金有价，信心无价。我们的信心来自党中央、国务院和自治区党委、政府及市委的坚强领导，来自学习实践科学发展观活动形成的强大动力，来自历届市委、市政府“打基础、谋长远”积蓄的发展能量，来自全市干部群众思进取、谋发展的决心和干劲。有信心就有迎接挑战、战胜困难的力量，就能够把广大干部抓经济工作的积极性、主动性、创造性充分调动起来，把投资者的投资热情焕发出来，把人民群众对经济发展的良好心理预期提振起来。只要我们坚定信心和决心，做好应对复杂经济形势和严峻考验的充分准备，就能够化压力为动力，化挑战为机遇，就能够赢得工作主动权。

2009年政府工作的总体要求是：以邓小平理论和“三个代表”重要思想为指导，深入学习实践科学发展观，全面落实党的十七大和十七届三中全会精神，贯彻落实自治区党委、政府的工作部署，把保持经济平稳较快发展作为首要任务，围绕加快推进工业化、城镇化，全面融入泛珠三角经济区和广西北部湾经济区，着力抓好强柴兴玉，全力建设广西现代农业示范市和非公经济示范市，立足扩大内需、强化投资拉动，深化改革创新、扩大开放合作，转变发展方式、增强发展活力，加强社会建设、保障改善民生，促进经济社会科学发展、和谐发展、跨越发展，以优异成绩迎接新中国成立60周年。

经济社会发展主要预期目标为:生产总值增长12%，全社会固定资产投资增长25%以上，财政收入增长12.3%，社会消费品零售总额增长20%。万元GDP能耗下降6.51%，二氧化硫排放总量削减0.98万吨，化学需氧量排放总量削减1.24万吨。工业增加值增长16%，外贸进出口总额增长7%，实际利用外资增长20%。城镇登记失业率控制在4.5%以内，城镇居民人均可支配收入增长12%，农民人均纯收入增长11%，人口自然增长率控制在10‰以内。居民消费价格指数涨幅控制在5%以内。

上述目标体现了加快发展的要求，有利于增强信心、稳定预期、促进发展。在不确定因素增多的形势下，实现上述目标需要比往年付出更大的努力。在实际工作中，我们将朝着更高的目标奋进，力争又好又快发展。

三、2009年主要工作部署

围绕今年经济社会发展目标，要着重在以下几个方面抓落实。

（一）千方百计扩大投资，保持投资的强劲拉动。把扩大投资作为促进增长的主要措施，强化项目投资促发展的理念，在全市范围深入开展“服务企业，推进项目年”活动，紧紧抓住项目建设这个经济发展的“牛鼻子”不放松，努力实现全市项目投资的新突破。

加快推进项目建设。今年，我市初步安排实施总投资3000万元以上重大建设项目418个（含续建和新开工项目），总投资784亿元，年内计划完成投资220亿元。建立完善项目建设协调督办机制，加快落实资金、用地和报批等工作，新开工一批项目。创造条件把今后几年开工的项目提前集中到今年开工建设。加快推进续建项目建设，积极配合抓好玉林至铁山港高速公路建设，加快龙潭至山口一级公路、城市电网改造和农网完善等重大基础设施和产业项目建设。

加强项目前期工作。列入今年前期工作的重大项目382个，项目总投资833亿元。建立项目前期工作经费投入机制，从今年起，市级预算将较大幅度增加项目前期工作经费投入。切实完善项目前期工作机制，建立和完善部门联席会议制度，健全项目前期工作目标责任制。抓好各项专项规划的编制，加强与自治区、国家有关部门沟通衔接，力争我市有更多重大项目列入自治区和国家规划项目，获得更多支持。切实做好项目储备，超前谋划一批重大项目，不断充实和完善项目库。优化办事流程，采取特事特办的方法，加快项目审批工作。

多渠道筹措建设资金。积极构建项目投融资平台，加大预算内资金投入，拓宽项目建设资金来源渠道。创新投资和项目建设资金管理机制，更多地利用市场的办法筹措建设资金。加强与银行及国家、自治区有关部门的联系对接，争取信贷资金、国债投资、国家预算内投资、自治区本级预算内基建资金及其他专项资金支持项目建设。

（二）以强柴兴玉为引领，加快新型工业化进程。坚持以工业化为核心战略和主导方向，继续发展壮大机械、水泥陶瓷、健康、服装皮革、电子信息等产业集群。继续举全市之力支持玉柴发展，通过玉柴的跨越式发展，带动玉林工业加快发展。

深入实施“强柴兴玉”战略。在项目规划、审批、用地、资金、用电用水以及人才引进等方面给予玉柴更大的支持和更优质的服务。今年，重点抓好36个总投资40亿元的“强柴兴玉”项目的建设。支持玉柴做强做大柴油机产业，进军汽车产业，进行多元化投资，建设国际性汽配生产、采购基地，不断延伸产业链，做大企业规模。充分发挥玉柴集聚辐射作用，引导关联企业本土化，逐步提高零配件的本土配套比例。积极筹建国家内燃机及零部件产品质量监督检验中心，加快玉柴工程研究院、玉柴企业博士后科研工作站和人才小高地建设，提升玉柴企业竞争力。积极鼓励玉柴参与国家标准制订，保持玉柴产品在同行业的技术领先地位。支持玉柴实施品牌战略，打造国内一流、世界知名品牌，不断增强“玉柴机器”的品牌影响力。加快市经济开发区、玉柴工业园、陆川北部工业区规划和建设，打造品牌工业园，推动玉柴及配套企业加速发展。

加快推进技术改造。抓住当前全球产业大调整和自治区扩大技改投资的机遇，加大更新改造力度，加快推进总投资17亿元的玉柴铸造中心等一批重大技改项目建设。加大财政对企业技改的支持力度，支持重点骨干企业进行技术改造，提高产品科技含量和企业效益。引导支持中小企业主动与大企业开展协作配套，加快技术和新产品开发，提高产品技术层次和市场竞争力。鼓励和扶持建立技术服务机构，为中小企业提供技术信息、技术开发、技术咨询培训和技术转让服务。

加强服务企业力度。结合开展“服务企业，推进项目年”活动，进一步强化为企业发展服务。针对国外市场需求疲软的状况，支持企业努力开发组织适销对路的产品生产，大力开拓国内外市场。采取“一企一策”，分类帮扶企业，努力帮助广大中小企业度过难关，加快发展。

（三）夯实农业发展基础，建设现代农业示范市。全面贯彻党的十七届三中全会、中央农村工作会议和中央1号文件精神，发挥海峡两岸（广西玉林）农业合作试验区的示范带动作用，加快建设现代农业和农业产业化示范市，促进农业持续增效、农民持续增收和农村经济持续发展。

大力构建现代农业产业体系。优化农产品区域布局，因地制宜发展特色产业。在保障粮食安全的基础上，积极发展优质稻、中药材、花卉、食用菌、马铃薯、香蒜、桑蚕、沙田柚等优势特色产品，加快农业标准化示范区建设；大力发展畜牧水产业，建设一批集约化、标准化、产业化的现代特色农产品生产基地和渔牧养殖模式示范基地。加快发展特色优势农产品加工业，规划建设农产品加工物流园区，提高农产品深加工水平。加强与台湾在良种培育和繁殖方面的合作，发展种子种苗产业，提升农业发展水平和效益。加强和改善粮食宏观调控，做好粮食直补收购和储备工作。用好农机购置补贴政策，实施“千乡万村现代农机装备推进工程”，加快农业机械化和农机产业发展。

加快推进农业产业化。加大农业龙头企业培育力度，完善信贷担保、贷款贴息、用地用电等扶持政策。从今年起，市本级每年安排100万元农业产业化专项经费，安排50万元农业品牌申报经费，重点支持龙头企业技术创新、品牌创建和建设特色农产品标准化生产基地。积极发展农村合作经济组织，带动农业规模发展，促进农业产业化。

加强农业基础设施建设。抓住中央大幅度增加对“三农”投入的机遇，大力实施以水利基础设施为重点的农业基础设施建设大会战。突出抓好以病险水库除险加固、农村饮水安全为重点的民生水利工作，完善农业灌溉体系，提高水资源综合利用率。继续实施通乡通村柏油路、乡镇客运站等项目，抓好生态家园和集约化养殖场大中型沼气工程建设。进一步完善新农村建设规划，扎实推进新农村建设。

完善农村社会化服务体系。加快建立新型农技推广服务网络，建立完善疫病防控、植物保护、产品质量安全、农村信息等服务体系。继续推行村村派驻科技特派员制度。加快农业研发和技术推广应用，促进农业产业结构调整。支持科研单位、供销社、农民专业合作社、龙头企业等提供与农业生产密切相关的技术咨询、技能培训和生产经营服务。积极探索信息服务进村入户的途径，加快农村信息化进程。

努力增加农民收入。全面落实良种补贴、农资综合直补等强农惠农政策。千方百计减轻农民负担。加大培育劳务产业力度，整合各类培训资源，有针对性地开展城乡劳动力职业技能培训，提高农民就业和创业能力。重视解决返乡农民工培训、就业、创业问题。

（四）积极扩大消费需求，加快发展现代服务业。贯彻落实促进消费的各项政策，稳定消费预期，培育消费热点，推动消费持续走旺。

积极扩大消费需求。通过完善社会保障体系、扩大就业、稳定增加城乡居民收入等措施，增强居民消费信心。进一步挖掘消费潜力，增强消费需求特别是居民最终消费需求对经济增长的拉动力。稳定扩大住房、汽车等大宗消费，大力发展社区物业、家政等服务性消费，积极推进旅游、文化、健身和网络等热点消费，促进消费结构升级。研究制定鼓励消费的具体措施，发展和完善消费信贷，优化消费环境。抓住国家启动“家电下乡工程”的契机，深入实施“万村千乡市场工程”，建设一批农村农家便利店、乡镇超市，推进农村网点超市化、货物流通配送化，大力开拓农村消费市场，促进农民消费。

搞活商贸经济。坚持突出特色、适度集中布局，加强玉林城区商业网点规划建设和提升改造，整合提升现有市场。鼓励多种经济成分参与市场建设开发，继续推进银丰国际中药港、宏进农副产品批发市场等一批重大市场及物流项目建设，逐步形成以玉林城区专业批发市场为龙头，各县（市、区）专业批发市场为骨干，重点乡镇农贸市场为补充的市场体系。创新商贸业结构、体制和经营方式，引进培育一批大型现代商贸企业，全面提升商贸业档次和水平，打造连接“两广”、辐射东南亚的桂东南区域性商贸物流枢纽。

加快发展现代服务业。加快商贸物流网络建设，大力发展电子商务，不断创新市场商贸手段。依托大型批发市场，整合仓储、货运资源，强化物流平台建设，构建农副产品和日用小商品物流配送中心，发展现代物流产业。大力发展旅游业，建设旅游强市。加快发展信息服务、现代会展以及文化产业等新兴服务业，推动服务业上新台阶。

（五）加快城市建设步伐，提高城市综合竞争力。创新城市规划理念，推动城市科学发展。积极推进城市新区建设，扩张城市规模，着力改善城市人居环境和经济承载力，提升城市综合竞争力。

深入推进玉-北-福一体化。加快二环路南段等一批城市路网建设，打通连接玉东新区和江南新区的城市通道，拉开城市框架，加快新区建设步伐，做大做强玉林中心城区。以文化公园、文化会议中心、教育集中区、城东汽车站、妇女儿童活动中心等项目为重点，加快玉东新区建设发展。精心组织完善江南新区的商业项目，打造玉林现代服务业平台。以玉柴工业园区建设为带动，加快建设玉柴新区。

争创广西园林城市。力争成功申报广西园林城市。突出抓好迎宾大道街边公园、西郊公园、牛运岭公园、南流江两岸等10个项目建设。深入开展“城乡清洁工程”，持续开展全民义务植树绿化活动，全面提高城乡绿化水平，实现全市森林覆盖率55%。加速推进玉林城区水环境治理、垃圾收运系统、城市生活垃圾处理二期、污水处理厂二期等18个项目建设，不断完善城市服务功能。推进老城区路网改造，加快小街小巷硬化，进一步改善城市交通条件。加快“数字城管”建设，提高城市管理水平。统筹城乡发展，突出地方特色，加快城镇风貌改造，加强城乡自然、文化遗产保护，提高城镇品位。

稳健发展房地产业。把发展房地产业作为扩大内需的重要手段，鼓励上马大型高品质的房地产项目，使房地产业继续保持旺盛的发展态势。重点抓好奥园、盛世江南、银丰·江南新城等29个房地产项目，力争完成投资40亿元。继续抓好廉租住房建设和公房改造。

（六）坚定不移扩大开放，积极参与北部湾开发。以开放合作拓展发展空间，打造泛北部湾中小企业名城。

构建通江达海的交通网络。加快玉林至铁山港高速公路、龙潭至山口公路和洛湛铁路玉林段场站建设；配合做好玉林至北海铁路的前期工作，争取尽快开工建设；争取玉林至钦州高速公路尽快列入规划并开展前期工作；积极争取荔浦至铁山港高速公路荔浦至玉林段列入计划开工建设，与北海市合作加快推进铁山港东岸码头规划建设。加快完善等级公路网。加快玉林至陆川、玉林至福绵一级公路等干线公路建设，规划建设一批县际路、路网路和出口公路。抓好北流至宝圩一级公路改造，把陆川至盘龙一级公路改造列入规划。

加快龙潭产业园建设。进一步修编完善产业园规划，加快推进路、水、电和标准厂房、商贸物流等基础设施建设。抓好入园项目落地工作，加快推进再生资源进口加工利用等重大项目建设，做强做大广西银亿等一批重点企业，形成产业优势，把龙潭产业园打造成为北部湾经济区有色金属加工制造基地、再生资源加工利用基地、仓储物流基地。

建设广西东部产业转移示范区。自治区已把支持桂东地区基础设施建设列入今年为民办实事工程。要把握机遇，落实政策和资金，积极开展广西东部产业转移示范区基础设施建设,加快推进园区征地、“三通一平”等基础设施和标准厂房建设，完善园区招商引资机制，抓好项目落地，进一步改善承接产业转移的环境。

全面拓展区内外合作交流。配合办好第六届中国-东盟博览会，全力办好第六届玉博会，不断完善区域合作平台。积极开展面向珠三角、长三角和港澳台等地区的招商推介活动，推进区域合作向深层次、宽领域发展。

提高外贸出口和利用外资水平。深入实施开放带动战略，以大开放促进大发展。进一步转变外贸发展方式,优化外贸出口结构，扩大高附加值产品出口;找准切入点，积极扩大对东盟的出口。认真落实出口退税政策，积极发展出口信用保险，扩大对中小外贸企业的融资担保;完善外贸工作协调机制，及时协调解决出口企业的困难，努力保持外贸出口稳定增长。积极扩大利用外资规模，继续实施大招商、招大商。坚持品牌引领，着力引进关联度大、带动力强的世界500强、中国500强企业；实施产业链招商、“百企入桂”品牌招商和定点定向招商引资活动。积极推进招商引资项目大兑现，全面落实招商引资优惠政策，提高引进项目的合同履约率、资金到位率和开竣工率。

（七）促进全民创新创业，建设非公经济示范市。把发展非公经济作为加快经济发展的重要着力点和重大举措，毫不动摇地鼓励、支持、引导非公经济加快发展。

开展全民创新创业活动。完善和落实激励全民创新创业的政策，加强职业技能培训、创业培训和农村劳动力转移就业技能培训，完善创业服务体系，加大创业扶持力度，鼓励各类人才自主创业，扶持农民种养创业，支持个体业主二次创业。加快完善创业载体和平台。引导非公企业围绕大企业、大产业配套发展，走专、精、特、新和集约化、规模化发展道路；加快建设特色产业园区，引导非公企业集聚发展；鼓励非公企业合资合作，推动产业对接互补，加快创业孵化体系建设，建设创业型城市、创业型县区、创业型乡镇。

拓宽非公经济融资渠道。加快建立中小企业信用评价体系，鼓励商业银行加大对非公企业的信贷支持力度，采取授信办法，优先保证诚实守信、运行良好的民营企业发展资金需求。进一步健全融资担保体系，鼓励民间资本参与投资设立中小企业信用担保机构，为非公企业提供融资担保服务，增强非公企业融资能力。

推动非公经济自主创新。引导非公企业建立规范的法人治理结构，创新管理模式，提高决策和管理水平。鼓励企业采用国际先进标准组织生产。积极扶持科技示范企业和高新技术企业。鼓励非公企业建设技术中心，加大技改投入，实施品牌发展战略和技术标准战略，提高企业技术创新和产品创新能力。对自主创新和品牌培育成绩显著的非公企业和个人给予表彰。

（八）统筹推动协调发展，增强可持续发展能力。推进节能减排，统筹人口、资源、环境协调发展，增强可持续发展能力，建设资源节约型、环境友好型社会，实现人与自然和谐发展。

抓好节能减排工作。强化节能减排目标责任制。着力推进城镇污水垃圾处理设施和工业园区集中式污水处理设施建设,确保陆川、北流污水处理厂在年内建成投入运行，力争博白、容县、兴业污水处理厂在年底前竣工。加快市医疗废物处置中心建设，争取在年内竣工投入试用。加强重点工业企业生化治污工程建设。加快淘汰立窑水泥、小造纸等落后产能，抓好建材、造纸行业年耗能5000吨标准煤以上的企业节能降耗工作。开发和推广建筑节能技术和节能照明产品。

加强人口计生工作。加大宣传教育力度，进一步转变群众生育观念；加强县乡服务站所基础建设，巩固提高基层计生管理和服务能力；强化人口出生监控工作，严格控制政策外生育，稳定低生育水平；完善计划生育奖励扶助制度，探索建立计划生育家庭养老保障制度和财政贴息小额贷款扶持制度；建立依法管理、村民自治、优质服务、政策推动、综合治理的人口和计划生育工作新机制，改善人口结构，提高人口素质。

严格保护耕地资源。制定并严格实施全市土地利用总体规划，坚守422.65万亩耕地红线目标。坚持占用耕地补偿制度，探索建立耕地保护基金和分类分级保护激励机制，建立完善耕地保护目标责任制。

切实保护和改善生态环境。抓好饮用水源地保护，重点加强南流江、清湾江、绣江、九洲江等水功能区保护。实施农村环境综合整治，推行生态养殖，大力发展沼气等清洁能源。探索建立生态公益林补偿机制，推进大容山、天堂山自治区级自然保护区建设，加强水源涵养林、水土保持林等生态公益林的保护和建设。开展重点区域土壤治理试点工作，加强地质灾害防范治理。抓好水土流失的治理、监测和执法监督，推进清湾江河道和南流江福绵段治理。加快生态工业园区试点示范建设，大力推广清洁生产。强化污染源监控和执法监督。

（九）继续深化改革创新，着力增强发展活力。加强机制体制建设，推进自主创新，破解发展难题，积蓄发展能量，增强发展活力。

深化国资监管体制和国有集体企业改革。加强跟踪指导，加快推进国有企业改革，完善国有资产管理体制，规范国有企业改制和产权转让，提高国有资产运营效益。支持民间资本参与国有企业改革。加快城镇集体企业改革步伐。

积极推进农村各项改革。稳定和完善农村基本经营制度，依法、自愿、有偿推进农村土地承包经营权流转，加快农村规模化产业化经营步伐。稳步推进农村集体林权制度改革。加快农村小型水利管理体制改革，鼓励社会资金投入水利建设。推进基层农技管理体制改革和农村金融改革，引导更多信贷资金和社会资金投向农村。

深化财税、金融、投资体制改革。深化部门预算、国库集中支付和政府采购制度改革等支出改革，继续推进规范津贴补贴改革，深化非税收入管理改革，狠抓增收节支。积极配合实施增值税转型改革和燃油税费改革，认真落实各项税收改革，强化税收征管工作。强化政银企合作，继续支持金融部门机构改革，稳步推进金融机构综合经营试点和政策性农业保险试点。完善企业投资备案制，大力推行代建制。加强和改进价格监管，抓住机遇推进资源性产品价格改革。

加快自主创新体系建设。围绕壮大优势产业，加大技术创新、产品创新和管理创新力度，增强企业和产业竞争力。积极参与自治区重大产业研发中心的建设，全力争取内燃机、日用瓷、中成药等自治区工程技术研发中心落户我市企业。实施人才强市战略，创新完善高层次人才引进和培养使用机制，优化人才发展环境。深化公务员队伍管理和人事制度改革，逐步实行事业单位岗位设置管理。培育和发展科技企业孵化器、创业风险投资机构等创新组织，营造良好创新环境。

（十）切实保障改善民生，让群众共享发展成果。把发展经济与改善民生相结合，加大民生投入力度，切实改善和保障民生，让广大群众共享更多发展成果。全年财政安排保障和改善民生资金7.83亿元。

千方百计稳定和扩大就业。今年，市财政安排就业专项资金120万元，城镇新增就业2.09万人，农村劳动力转移就业新增4.3万人。实施积极的就业政策，全面落实就业促进法和税收优惠、小额担保贷款等鼓励创业的政策措施，稳定职工队伍，大力开发就业岗位，促进充分就业。统筹做好大中专毕业生、城镇新增劳动力、城镇登记失业人员、返乡农民工和农村劳动力转移就业工作。加强公共就业服务体系建设，加强对企业劳动关系的指导和服务，促进劳动关系和谐稳定。

为民办好十件实事。继续集中人力、财力和物力，进一步调动受益群众和社会各界的积极性，办好、办实与民生密切相关的十件实事：一是实施库区移民和革命老区基础设施建设工程。对全市大中型水库库区实施村屯道路、通水、通电、教育、卫生和破旧危房进行改造。市本级安排210万元专项资金，对革命老区村实施村道畅通、小型水利、通电、学校危房改造等工程。二是实施教育惠民工程。市财政安排1953万元用于职业教育攻坚。市本级安排80万元奖助学金，继续资助考上大学的贫困家庭子女和普通高中家庭经济困难学生。对农村义务教育阶段经济困难寄宿生补助生活费，免费提供全部农村义务教育学生教科书，免除城市义务教育阶段学生学杂费。三是实施城乡医疗惠民工程。全面推进新型农村合作医疗制度建设。在自治区财政补助的基础上，市财政再安排2814万元，使农民参合率达到90%以上。推进城镇居民基本医疗保险试点，按照每人每年30元的标准补助，力争参保居民达到应保人数80%；全面建立城乡医疗救助制度，对参加城市居民基本医疗保险的城镇低保对象每人每年补助20元以上，全额资助五保对象、适当资助农村低保对象参加农村合作医疗。四是实施乡村文化体育建设和村村通广播电视工程。年内建成48个乡镇综合文化站，为16个乡镇综合文化站、280个村委会配置文化活动设备，在100个村屯建设农民健身篮球场，在144个村屯建设农家书屋，开展送书、送戏、送电影下乡。完成738个20户以上已通电自然村的村村通广播电视建设任务。五是实施廉租房建设工程。继续完善廉租住房制度，缓解城市最低收入困难家庭的住房困难问题。六是实施乡镇敬老院建设工程。新建10所乡镇敬老院。七是实施农村人口饮水安全工程。解决20.5万农村人口饮水安全问题。八是实施农村沼气池建设工程。新建沼气池14000座。九是实施县乡计生服务站所建设工程。市财政安排200万元，加强县乡两级计生服务站所基础设施建设。十是实施“万村千乡市场建设工程”。年内再建200个农家店。实施家电下乡，对农民购买彩电、冰箱、洗衣机和手机等给予资金补助。

稳步提高低收入人群收入。完善收入分配政策，提高职工工资水平，稳步提高社会保险对象待遇、企业退休人员基本养老金水平和优抚对象生活补助标准。加大城乡最低生活保障和困难救助补助资金投入，完善城乡居民最低生活保障制度和困难群众临时救助制度，提高低保保障标准。进一步落实农村五保户供养政策，提高月人均补助标准。按照国家新扶贫标准，扩大扶贫范围，加大扶贫投入，努力减少贫困人口。全面启动第三批贫困村的“整村推进”扶贫，加大基础设施、产业化扶贫和扶贫培训力度，争取实施贫困村危房改造工程。

加快完善社会保障体系。今年，继续扩大社会保险覆盖面，五项社会保险资金投入8.5亿元。进一步规范企业职工基本养老保险基金预决算管理。做好事业单位养老保险工作，建立村干部养老保险制度，逐步提高村干部的待遇水平。完善城镇职工基本医疗保险制度、生育保险制度和工伤保险制度。提高医疗保险统筹层次，完善工伤保险制度，建立基本医疗保险、工伤保险市级统筹。全面推进城镇居民基本医疗保险。推进农民工参加工伤保险和医疗保险，稳步推进农民工养老保险。加快建立健全城乡医疗救助体系。加快推进职工帮扶中心建设。加强自然灾害应急救助管理，提高防灾、抗灾、救灾能力。

（十一）加快发展社会事业，促进经济社会协调发展。加大社会事业发展投入，促进社会事业全面繁荣。

优先发展教育事业。今年，全市财政安排教育支出21.3亿元，比去年增加5.87亿元。认真贯彻落实自治区“两基”巩固提高工作计划，进一步促进基础教育均衡发展。加大教育基础设施建设投入，在玉林城区玉东、江南新区分别开工建设1所小学，继续抓好农村中小学校舍维修改造长效机制项目和校舍标准化建设，推进中小学布局调整；实施初中校舍改造工程、中职基础能力建设、特殊教育学校建设项目；加强示范性高中建设，争取新立项省级示范性高中1～2间，提高高中阶段教育普及率。大力支持玉林师院申请立项建设为硕士学位授予单位，服务好玉林师院发展。深入推进职业教育攻坚，启动市县职教中心建设工程。鼓励和规范发展民办教育。落实教育资助政策，保障贫困家庭学生、农民工子女、留守儿童平等接受义务教育。深化教育教学改革，强化教育督导考核，全面推行素质教育。加强教师队伍建设，提高教师素质。着力解决农村师资力量不足问题。

大力发展文化事业。今年，全市财政安排文化、体育和传媒支出7510万元，比去年增加1364万元。发展公益性文化事业，加快建设公共文化服务体系，积极推进科普工作。建设市文化中心、报业中心、文化公园、博物馆、乡镇综合文化站等项目。加强社区和乡村文化设施、广播电视村村通、文化信息资源共享等文化工程建设，支持影剧院等文化场所建设，引导民间文艺组织健康发展，促进群众文化娱乐活动开展。鼓励文艺创作，打造更多更好的精神文化产品。抓好庆祝建国60周年等节庆的文化活动。继续做好第三次全国文物普查工作，加强文物和非物质文化遗产保护。抓好“扫黄打非”工作，净化出版物市场，整治低俗淫秽网络文化。深化文化体制改革，大力培育发展文化产业。着力筹划在我市举办的全区第12届少数民族传统体育运动会，加强农村体育设施建设，加快发展体育产业，促进体育与经济社会协调发展；积极推进全民健身计划，提高竞技体育水平。

加快发展卫生事业。今年，全市财政安排医疗卫生支出3.39亿元，比去年增加1.2亿元。重点抓好市第一人民医院门诊大楼等项目建设。全面开展新型农村合作医疗，完善农村三级卫生服务网络和城市社区卫生服务体系。做好广西卫生城市复核工作。加强医德医风和医疗卫生人才队伍建设，提高医疗卫生服务水平。加大传染病预防控制力度，提高突发公共卫生事件应急处置能力。

（十二）加强民主法制建设，切实维护社会和谐稳定。

推进民主法制建设。完善民主决策机制，自觉接受人大法律监督、政协民主监督，认真办理人大代表议案、建议和政协委员提案，重视民主党派、工商联参政议政的作用。充分发挥工会、共青团、妇联等群团组织的桥梁纽带作用。坚持问计于民，力求使决策更科学、更符合群众意愿。推进法治政府建设，提高依法行政水平。加强行政监察、行政复议和审计等方面工作，全面落实行政执法责任制、评议考核制和行政执法过错责任追究制，加强对行政权力运行的制约与监督，严肃查处行政不作为和乱作为。

加强安全生产和食品药品监管。强化企业安全生产主体责任和各级政府的监管责任，加强重点行业、重点领域的安全生产，加强安全生产培训和宣传教育，全面排查和治理重大安全隐患，依法严厉查处各类安全生产违法违规行为，防范和遏制重特大安全事故，进一步减少事故总量。实施食品药品放心工程，加强农村药品“两网”建设，健全食品药品安全责任体系，强化源头监控和市场监管，严肃查处产品质量安全违法违规案件，构建保障产品质量安全的长效机制，确保群众饮食和用药安全。

切实维护社会和谐稳定。加强社会矛盾纠纷排查调处和预警体系建设。加强和改善信访工作，继续开展“大接访”活动，畅通民意表达渠道，依法及时妥善处理群众反映的突出问题。继续加强基层司法所建设。加强征地拆迁、农村土地、水利、山林纠纷等影响社会稳定突出问题的排查化解工作，从源头上解决影响稳定的突出问题。深入开展平安玉林、平安县区创建活动，健全社会治安防控体系，依法防范和打击违法犯罪活动，推动社会治安综合治理和平安建设工作的均衡发展。切实加强和改进以人口管理为重点的社会管理。完善应急预案和体制机制，有效预防和妥善处理各类突发性群体事件。

做好支持部队和国防建设工作。积极支持驻玉部队、民兵预备役和武警部队建设，抓好国防动员、人民防空和后备力量建设。广泛开展国防教育，深入开展双拥共建活动。落实军人优抚政策，做好军队转业干部、复员退伍军人安置工作。

加强统计工作，搞好第二次全国经济普查。做好“十二五”规划的前期工作。继续做好外事侨务、对台事务、民族宗教、防震、老龄、残疾人、妇女儿童、档案、修志、气象、水文、无线电管理等工作。

新形势、新任务对政府工作提出了更高要求，必须加强政府自身建设。要认真贯彻落实上级和市委的决策部署，确保政令畅通。加强市县依法行政，深化行政管理体制改革，理顺部门职责分工，推动政府职能转变到位。继续减少和规范行政审批，加强和改善经济调节和市场监管，更加注重履行社会管理和公共服务职能。进一步健全重大工作部署督办、机关绩效考评、行政效能监察等激励监督机制，推进政务公开，加强电子政务建设，切实减少“文山会海”,促进机关工作高效运转，致力打造优质政务环境。深入推进政府廉政建设，大力弘扬艰苦奋斗作风，坚持“两个务必”，反对和制止奢侈浪费。充分发挥市场在资源配置中的基础性作用，通过市场机制公开、公平配置资源。继续全面落实党风廉政建设责任制，注重从源头上预防和治理腐败。切实解决群众反映强烈的问题，坚决纠正损害群众利益的不正之风，严厉查处各类违法违纪案件，营造风清气正谋发展的良好氛围。

各位代表！做好今年各项工作，挑战与机遇并存，困难与希望同在，任务艰巨，责任重大。让我们高举中国特色社会主义伟大旗帜，以邓小平理论和“三个代表”重要思想为指导，在自治区党委、政府和市委的领导下，深入贯彻落实科学发展观，坚定信心，团结奋进，共克时艰，锐意进取，努力开创建设富裕文明和谐新玉林的新局面，以优异的成绩迎接新中国成立60周年!
